# IL‐1Ra deficiency accelerates intervertebral disc degeneration in C57BL6J mice

**DOI:** 10.1002/jsp2.1201

**Published:** 2022-04-23

**Authors:** Ganesh Swamy, Paul Salo, Neil Duncan, Frank Jirik, John Matyas

**Affiliations:** ^1^ Cumming School of Medicine McCaig Institute of Bone and Joint Health University of Calgary Calgary Alberta Canada; ^2^ Department of Surgery Cumming School of Medicine Calgary Alberta Canada; ^3^ Department of Civil Engineering Schulich School of Engineering Calgary Alberta Canada; ^4^ Department of Medicine Health Research Innovation Centre Calgary Alberta Canada; ^5^ Department of Comparative Biology & Experimental Medicine Faculty of Veterinary Medicine University of Calgary Calgary Alberta Canada

**Keywords:** IL‐1ra deficiency, intervertebral disc, mouse strain

## Abstract

The expression of Interleukin‐1ß (IL‐1ß) and its antagonist and Interleukin‐1 receptor antagonist (IL‐1Ra) are correlated with greater human intervertebral disc (IVD) degeneration, suggesting that elevated IL‐1β activity promotes disc degeneration. Many in vitro studies support such a mechanistic relationship, whereas few in vivo investigations have been reported. The present study tests the effect of increased IL‐1β activity on intervertebral disc in mice with an IL‐1Ra gene deletion.

IL‐1Ra−/− mice and wild‐type (WT) C57Bl6J mice were examined at 3 and 12 months of age. Caudal IVD segments were evaluated for disc degeneration by histopathology, functional testing, and inflammatory gene expression relevant to IL‐1β pathways. To test differences in injury response, pinprick annular puncture was performed on IL‐1Ra−/− and WT mice and evaluated similarly.

IL‐1Ra−/− IVDs had significantly worse histopathology at 3 months compared to WT controls, but not at 12 months. IL‐1Ra−/− IVDs exhibited significantly more viscous mechanical properties than WT IVDs. qPCR revealed downregulation of inflammatory genes at 3 and 12 months in IL‐1Ra−/− IVDs, with concomitant downregulation of anabolic and catabolic genes. Annular puncture yielded no appreciable differences between 2‐week and 6‐week post‐injured WT and IL1‐Ra−/− IVDs in histopathology or biomechanics, but inflammatory gene expression was sharply downregulated in IL‐1Ra−/− mice at 2 weeks, returning by 6 weeks post injury.

In the present study, IL‐1Ra deletion resulted in increased IVD histopathology, inferior biomechanics, and transiently decreased pro‐inflammatory cytokine gene expression. The histopathology of IL‐1Ra−/− IVDs on a C57BL/6J background is less severe than a previous report of IL1Ra−/− on a BALB/c background, yet both strains exhibit IVD degeneration, reinforcing a mechanistic role of IL‐1β signaling in IVD pathobiology. Despite a pro‐inflammatory environment, the annular puncture was no worse in IL‐1Ra−/− mice, suggesting that response to injury involves pathways other than inflammation. Overall, this study supports the hypothesis that IL‐1β‐driven inflammation is important in IVD degeneration.

## Graphical abstract

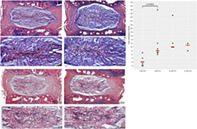

**Key Messages**

- IL‐1Ra −/− mice on the C57BL/6J background had increased and progressive histopathological degeneration between 3‐ and 12‐months of age.
- IL‐1Ra−/− IVDs undergo progressive functional degeneration, particularly in viscous biomechanical functional properties versus WT IVDs.
- At 3 months of age, IL‐1Ra−/− IVDs had sharply downregulated expression of genes encoding proteins involved in inflammation and matrix proteolysis compared to WT controls, suggesting co‐regulation of inflammatory and proteolytic gene expression pathways by IL‐1.
- The relatively modest histopathological differences in IL‐1Ra−/− C57BL/6J mice compared to previous reports of IL‐1Ra −/‐BALB/c mice suggest that variability of IVD degeneration phenotype might be accounted for by genetic differences in innate immunity among mouse strains.

## 1 INTRODUCTION

Intervertebral disc (IVD) degeneration is ubiquitous in humans and common in animals, with age being the most predictive risk factor.^1^, ^2^ Although the pathogenesis of IVD degeneration and back pain is incompletely understood, inflammation plays an influential role in the degenerative cascade. Low‐grade chronic inflammation is reported to be a feature of aging in humans (termed “inflamm‐aging”^3^), which ostensibly links maladaptive aging and IVD degeneration.^4^ While several cytokines have been implicated in inflamm‐aging, the IL‐1 axis is implicated both in organism physiological aging^5^ and in IVD aging through its second messenger system NF‐kB.^6^

The pro‐inflammatory cytokine IL‐1β is suspected to be an important mediator of IVD degeneration in vivo, as it is elevated in degenerate discs and is a powerful stimulus of matrix catabolism in the IVD in vitro and organ culture.^7^ Hence, IL‐1β represents a promising molecular target for modulating IVD structure and function. Indeed, a mechanistic link between IL‐1β and human IVD degeneration is supported by ex vivo investigations where IL‐1β and its endogenous receptor antagonist, IL‐1Ra, are expressed at much higher levels in degenerated and herniated human IVDs,^8^ which overexpress various pro‐inflammatory cytokines.^9^, ^10^ Increased expression of IL‐1β is potentially harmful, as it is known to upregulate matrix proteolytic enzymes that can degrade the matrix of the IVD, such as MMPs and ADAMTS‐4 and ‐5.^11^ In addition, IL‐1β activity itself promotes the expression of other pro‐inflammatory genes including TNF‐α and NF‐kB, both of which affect IVD cell function and survival.^12^ While previous ex vivo investigations link IL‐1β and IVD degeneration circumstantially, the precise role of IL‐1β in vivo remains obscure and underexplored.

To evaluate the mechanistic contribution of IL‐1β to skeletal homeostasis in vivo, several mouse models have been developed. In particular, deletion of IL‐1 Receptor Antagonist (IL‐1Ra), an endogenous competitive antagonist to IL‐1β, has been studied mainly in the context of inflammatory arthritis,^13^, ^14^ and also IVD pathobiology.^15^ In these models, deleting the IL‐1 receptor antagonist effectively *promotes* unchecked IL‐1 activity. The degree of development of inflammatory arthritis in IL‐1Ra−/− mice depends on background strain: spontaneous joint inflammation develops in BALB/c mice, but not in C57Bl/6.^16^ Previous work revealed spontaneous and severe IVD degeneration in BALB/c IL‐1Ra−/− mice shortly after birth, with further destruction and matrix mineralization. The primary aim of this study was to document the structure and function of the IVD in a systemic IL‐1Ra knockout mouse on the less inflammatory C57BL/6 background.^17^, ^18^

Annular injury models are commonly used to induce rapid IVD degeneration. Sobajima et al.^19^ documented a 3.5‐fold increase in IL‐1β gene expression at 3 weeks post injury in the NP of rabbit lumbar IVDs, along with a 2‐fold increase in MMP‐3 and iNOS gene expression. By 6 and 12 weeks, the same genes had been downregulated by −0.2 to −0.4‐fold, followed by a dramatic increase 24 weeks post injury (+4.8‐fold increase in IL‐1β).^19^ The initial spike in IL‐1β within the 1st week, and subsequent re‐normalization, has been confirmed at the gene expression and protein expression level in whole rodent IVDs (not only the NP).^20^, ^21^, ^22^ The post‐injury spike in IL‐1β gene expression is generally paralleled by spikes in other pro‐inflammatory mediators, including IL‐6, TNFα, and NF‐kB.^20^, ^23^ Given the role of IL‐1ß in this model, we chose to carry out annular injuries, hypothesizing that the IL‐1Ra−/− mouse would have accelerated structural and functional IVD degenerative changes.

## 2 METHODS

### 2.1 Mouse husbandry and grouping

All investigations were carried out with the approval of the Animal Care Committee at the University of Calgary (Protocol M11008). IL‐1Ra +/− mice heterozygote breeding pairs on a C57BL/6J background were purchased from Jackson Laboratories (Stock number 004754) and bred. Mice were housed in the University of Calgary double‐barrier facility on a 12‐h light cycle and given access to commercial chow and water ad libitum.

For investigation of age‐related changes in the IL‐1Ra−/− mouse (or uninjured IVDs), homozygous IL‐1Ra−/− (knockout or KO) and wild‐type (WT) mice (*n* = 11 each) were allocated randomly to two age groups: 3 and 12 months of age (*n* = 44 total). For the annular injury model, 10–12‐week‐old mice were selected, and 11 WT and 11 IL‐1Ra−/− mice were selected for injury at each of the two endpoints of 2 and 6 weeks (*n* = 44 total).

### 2.2 Annular injury model

After induction of general anesthesia (1–3% isoflurane and 1% oxygen), mice were administered antibiotic prophylaxis with enrofloxacin at 5 mg/kg at induction and 12 h post surgery. For analgesia, meloxicam, a non‐steroidal anti‐inflammatory, was administered at induction at 0.4 mg/kg, and another single dose was given 12 h post surgery. Skin preparation was carried out using a 2% chlorhexidine gluconate and 70% isopropyl alcohol mixture (3M SoluPrep).

Mice were positioned supine, and the caudal 4–5 intervertebral disc level was approached by an incision 2.5 cm distal to the base of the tail. Using the operating microscope, the interval between the mid‐ventral caudal artery and the right ventral tail flexor tendon was developed. Under direct visualization, a 33‐gauge lancet (BD Ultra‐Fine 33G lancet, Becton Dickinson) was introduced into the IVD to a depth of 1 mm, held for 5 s, and then withdrawn. The skin was re‐approximated, and wound closure was with cyanoacrylate glue (3M Vetbond).

### 2.3 Euthanization and tissue sampling—Uninjured IVDs

Mice were euthanized by CO_2_ inhalation, weighed, and the length of the tail and torso were measured with Vernier calipers (accurate to 0.05 mm; Mitutoyo Canada). To confirm genotypes postmortem, the terminal 0.5 cm of the tail was processed for PCR genotyping using primers recommended by Jackson Laboratories.^24^

Caudal IVDs were dissected under an operating microscope using a #11 scalpel. The C3 and C4 vertebrae, with the intervening C3‐4 caudal disc, were first removed from the tail and kept moist. The C5‐6 and C6‐7 IVDs were excised for snap freezing at −80°C in liquid nitrogen. IVD segments were prepared for RNA extraction by carefully removing overlying soft tissues from the IVD. Next, a sharp cut was made with a scalpel to cut the vertebra proximal to the peri‐discal physes, leaving a thin wafer of epiphyseal bone. The IVD segment was snap frozen at −80°C in liquid nitrogen.

The C3‐4 IVD was then isolated for non‐destructive biomechanical testing, immediately followed by fixation and subsequent histological processing. These slides were then used for histological classification and immunohistochemistry.

To prepare the C3‐4 IVD segment for biomechanical testing, the prezygapophyses and transverse processes were sharply trimmed, and the vertebra was cut sharply at mid‐diaphysis. A modified pin vise (McMaster‐Carr 8455A18 Pin Vise) was used for mounting bone‐disc‐bone specimens for biomechanical testing, after careful trimming of the ends of the vertebrae, as described by Elliot and Sarver.^25^

### 2.4 Euthanization and tissue sampling—Injured IVDs

Injured IVDs were harvested after CO_2_ inhalation. For 5 mice from each group, the injured IVD was used for biomechanical testing and histology. For 6 mice from each group, the injured IVD underwent gene expression analysis.

### 2.5 Biomechanical testing and analyses

A multi‐modal approach was used to test the time‐dependent, non‐linear mechanics of the IVD using a rapid protocol to measure its elastic and viscoelastic properties.^26^ Elastic measures were obtained at high loading rates and primarily reflect properties related to the solid matrix and NP pressurization. Dynamic measures (or viscous properties) describe differences between slow and fast loading rates and compare IVD properties more dependent on fluid flow (probably NP > AF).

To minimize desiccation while maintaining biomechanical properties, the bone‐disc‐bone motion segment was coated with a droplet of low‐viscosity silicone oil (Sigma‐Aldrich, Product Number 378321) (after Nicolle et al.^27^). Preliminary experiments determined that mouse discs lost less than 4.3% of wet mass at 15 min, the time needed to perform mechanical tests. IVD segments were mounted on a Bose ELF 3200 ElectroForce Test Instrument (ElectroForce Systems). Axial load was measured with an in‐line 250‐gram load cell (or 2.45 N).

Mechanical loading of caudal bone‐disc‐bone specimens was done using a cyclic waveform with 19 preconditioning cycles (± 0.45 N [or approximately 0.40–0.45 MPa] at 1 Hz) to ensure a steady state was reached, prior to experimental measurements made on the 20th cycle. After a pause at 0 N (22 s), a cyclic sine wave under load control between 0.02 N and 0.06 N (or between 0.02 and 0.05 MPa) compression was carried out at four different frequencies (0.05, 0.1, 1.0, and 10 Hz) as part of the dynamic mechanical analysis (DMA), after Bowles et al.^28^

The elastic properties were calculated at the 20th cycle by fitting the curve to a double sigmoid function (after Smit et al.^29^) using custom‐written MATLAB code (MathWorks, Natick MA). The neutral zone was defined by inflection points of the second derivative of the double sigmoid function. Given these bounds, a simultaneous linear regression of all zones (compression, neutral zone, and tension) was computed, and stiffness was derived for each zone (after Sarver and Elliott^30^). Data were judged acceptable when a goodness‐of‐fit exceeded 0.85.

Viscous properties were analyzed after transforming from time‐domain data to the frequency‐domain using a fast Fourier Transform.^31^ The cross power spectral density was calculated in MATLAB (MathWorks, Natick, MA), and phase difference was calculated in radians.^32^ Storage and loss moduli were calculated as a function of sine and cosine of the phase difference (δ) respectively, and tan δ was calculated as the ratio of loss‐to‐storage modulus. Whereas storage modulus and loss modulus describe the ability of the IVD to store and absorb energy respectively, tan δ describes the relative efficiency of a material to absorb energy and is independent of dimensions; a higher tan δ indicates more viscous behaviour.

### 2.6 Histopathological and immunohistochemical analyses

Murine IVDs were fixed in 10% neutral‐buffered formalin, demineralized in EDTA, and embedded in paraffin. Sections of 15–20‐um thickness were cut, and every other slide was stained with H&E, yielding about 20 slides (40 sections) per disc. The ORS Spine classification system^33^ was used to evaluate IVD degeneration in the mid‐sagittal section by the lead author.

IL‐1β protein expression was immunolocalized using a rabbit polyclonal anti‐IL‐1β antibody (Abcam ab9722). Spleen was sampled as a positive biological control for IL‐1β; omission of the primary antibody was used as a negative methodological control. Aggrecan (Santa Cruz SC25674), COL2 (Abcam ab85266), ADAMTS‐5 (Elabscience E‐AB‐15506), and MMP‐3 (Abcam ab43015) immunostaining were also performed.

Alternate slides were deparaffinized (Slide Brite, Biocare Medical, Pacheco CA) and rehydrated in a graded series of ethanols. For IL‐1ß, antigen retrieval was with Proteinase K (20 μg/ml for 30 min at 37°C), followed by distilled water washing. For the other antibodies, antigen retrieval was performed using 0.25% hyaluronidase/PBS (Sigma H6254) for 60 min at 37°C, followed by distilled water washing. Endogenous peroxidase blocking was performed with incubation with 3% hydrogen peroxide. Non‐specific antigen blocking was performed with normal goat serum (10%) in 0.1% Triton‐X for 2 h. Slides were incubated overnight at 4°C with primary antibody (1:100 dilution or 0.5 mg/ml), washed thrice in PBS, and incubated in secondary antibody (biotinylated goat anti‐rabbit IgG [HRP], Novus NB7160 at 0.6 mg/ml) at room temperature for 2 h. After washing, visualization was with 3′‐diaminobenzidine (DAB) (Dako K3467) for 10 min, and slides were counterstained with hematoxylin.

### 2.7 Gene expression analysis

RNA was extracted from IVD frozen sections after Lee et al.,^34^ wherein IVDs were embedded in a DEPC ice block and cut exhaustively in a cryostat at 20 um. About 0.5 ml of TRIzol (Invitrogen) was added and samples were stored at −80°C prior to RNA extraction. To further disrupt the tissue after thawing, the TRIzol mixture was passed through an 18 g or 21 g needle in a 1 cc syringe. Phase separation with 300 μl of chloroform was performed, followed by precipitation with 1 volume of isopropanol. Glycogen (at 5 mg/ml) was added to increase precipitation yield. SuperScript III First‐Strand Synthesis System (Invitrogen, CA) was used to synthesize cDNA. RNA (250 ng) was reverse transcribed using the random hexamer primers, and cDNA was stored at −20°C.

An assortment of 12 gene primers were selected from the MGH PrimerBank,^35^ relevant to IL‐1 and IVD biology (Table S1). PCR Primers were synthesized at the University of Calgary. Primer efficiency was calculated for each primer pair using a cDNA dilution series. Due to the limited amount of starting materials, we chose β‐2‐microglobulin as the single reference gene.^36^ Real‐time quantitative PCR was carried out using 25‐μl reaction volumes^35^: initial denaturation at 95°C for 10 min, followed by 40 cycles of 95°C for 15 s, 60°C for 30 s, and 72°C for 30 s, with a final extension period of 72°C for 1 min.

For each qPCR datum for uninjured IVDs, the mean adjusted quantification cycle (C'q) was derived from three separate mice. For each qPCR datum for injured IVDs, the C'q was derived from three biological replicates of two discs in the 2‐week group. As the RNA yield was too low in some extractions, the C'q was derived from two biological replicates of two discs in the 6‐week group. Change in RNA expression was quantified using an efficiency‐corrected analysis (after Hellemans et al.^37^). First, a ΔΔCq normalization against the reference gene (β‐2‐microglobulin) was performed. Next, data were normalized against the 3‐month WT group. Finally, values were converted back to a log_2_ scale (C'q) for ease of interpretation and graphical visualization.

### 2.8 Statistical analysis

Continuous biomechanical and qPCR data were analyzed for normality with Shapiro–Wilk test. If normally distributed, ANOVA and subsequent Bonferroni post hoc testing were used; if not, Kruskal–Wallis test was used. Histological grades were analyzed using the non‐parametric Kruskal–Willis and Dunn post hoc tests. To test our primary hypothesis, significance was evaluated between 3‐month IL‐1Ra−/− and WT and 12‐month IL‐1Ra−/− and WT mice.

For qPCR, given the sample size, and with *α* = 0.05, *β* = 0.8, and a standard deviation of 0.75 cycles, qPCR was powered to detect a 2.1 C'q difference between groups by ANOVA.^38^, ^39^

## 3 RESULTS

### 3.1 Uninjured IVDs

The numbers and distribution of mice in all groups are shown in Table 1. At 3 months, the mean torso length of IL‐1Ra−/− mice was shorter than WT mice (Table 1) and did not grow significantly longer with age. IL‐1Ra−/− mice had a significantly lower body mass compared to controls at 3 and 12 months of age; at 12 months, the average weight of the KO mice was half that of the WT mice.

**TABLE 1:** Morphometric measurements of C57BL/6J IL‐1Ra−/− and wild‐type mice by age

	3 months KO (*n* = 11)	3 months WT (*n* = 11)		12 months KO (*n* = 4)	12 months WT (*n* = 10)	
Torso length (mm)	80.4 (4.4)	85.8 (3.8)	*p* = 0.01	86.2 (4.6)	98.0 (8.8)	*p* = 0.004
Tail length (mm)	76.7 (1.8)	77.4 (2.0)		82.8 (2.3)	79.2 (10.7)	
Body mass (g)	19.7 (2.6)	23.3 (3.4)	*p* = 0.02	20.7 (2.3)	53.8 (10.3)	*p* = 0.01

The mortality rate in IL‐1Ra−/− was low up to approximately 6 months. After this time, however, IL‐1 Ra−/− mice were commonly euthanized earlier than planned due to failure to thrive (as evidenced by weight loss and lethargy in the absence of another diagnosis). Consequently, although groups had equal numbers at the beginning of the experiment, the number of mice in the 12‐month IL‐1Ra−/− group (*n* = 4) was lower than in the 3‐month IL1‐Ra−/− group (*n* = 11) (Table 2).

#### 3.1.1 Histopathological analysis—Uninjured IVDs

**FIGURE 1:**
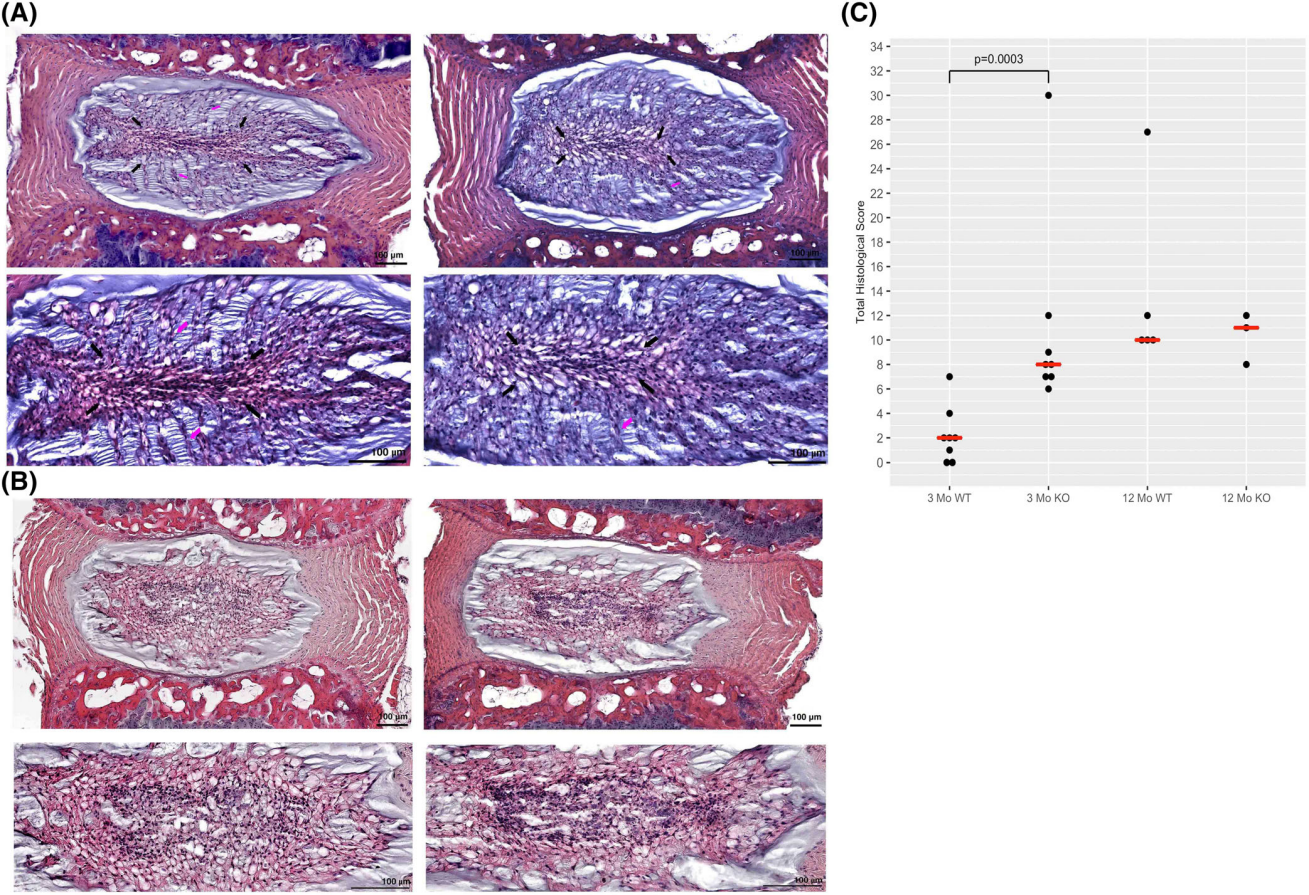
Uninjured IVDs stained with H&E. (A) 3‐month wild‐type mice mid‐sagittal histological images in 3‐month mid‐sagittal images, a consistent pattern is seen, with a central strip of flattened notochordal cells (black arrows). From the central strip, finger‐like extensions of notochordal cells (pink arrows) seem to extend radially. IL‐1Ra−/− NP regions exhibit altered cellularity with partial loss of notochordal cells. In addition, the central strip is larger, and the finger‐like extensions are less in number and cells. (B) 12‐Month Wild‐Type Mid‐Sagittal Images. At 12 months, we see an expansion of the central strip seen in 3‐month samples. Vacuolated notochordal cells surround the central area, which is filled with primarily smaller chondrocyte cells. In 12‐month mid‐sagittal images, the central strip is much narrower and contains areas of cellular debris. In addition, an identifiable border develops between peripheral matrix and notochordal cells. (C) Dot plot with median values showing histological score frequencies between IL‐1Ra−/− groups (KO) and WT groups. While there were occasional outliers with highly degenerative scores of 9 in either genotype, most scores clustered toward the lower end of the scale, indicating relatively normal structure in knockout IVDs. However, IL‐1Ra−/− scores were significantly higher at 3 months as per Kruskal–Wallis test (*p* = 0.0003)

Qualitatively, IL‐1Ra−/− IVDs exhibited a relatively normal morphological appearance at 3 months and were not appreciably different than those from WT mice (Figure 1A). In both groups, in mid‐sagittal sections of the NP, there was a central strip of small cells, accompanied by extensions of notochordal cells extending toward the periphery.

At 12 months, caudal IVDs of KO mice were more degenerate when compared to 12‐month WT mice. The expansion of the central zone of small cells was accompanied by less‐dense packing of the small cells (Figure 1B). Notably, the density of notochordal cells in KOs also appeared much diminished when compared to the IVD of 12‐month WT mice.

Three‐month IL‐1Ra−/− discs scored significantly higher (more pathology) compared to age‐matched WT mice (*p* = 0.0003 by Kruskal–Wallis test) (Figure 1C). At 12 months, the difference in scores was not significant (*p* = 0.1). In both 3‐ and 12‐month groups, the IL‐1Ra−/− mice differed from WT mice primarily in the cellularity and matrix composition of the NP, and less apparent differences in AF, EP, and boundary components.

#### 3.1.2 Biomechanical analysis—Uninjured IVDs

**FIGURE 2:**
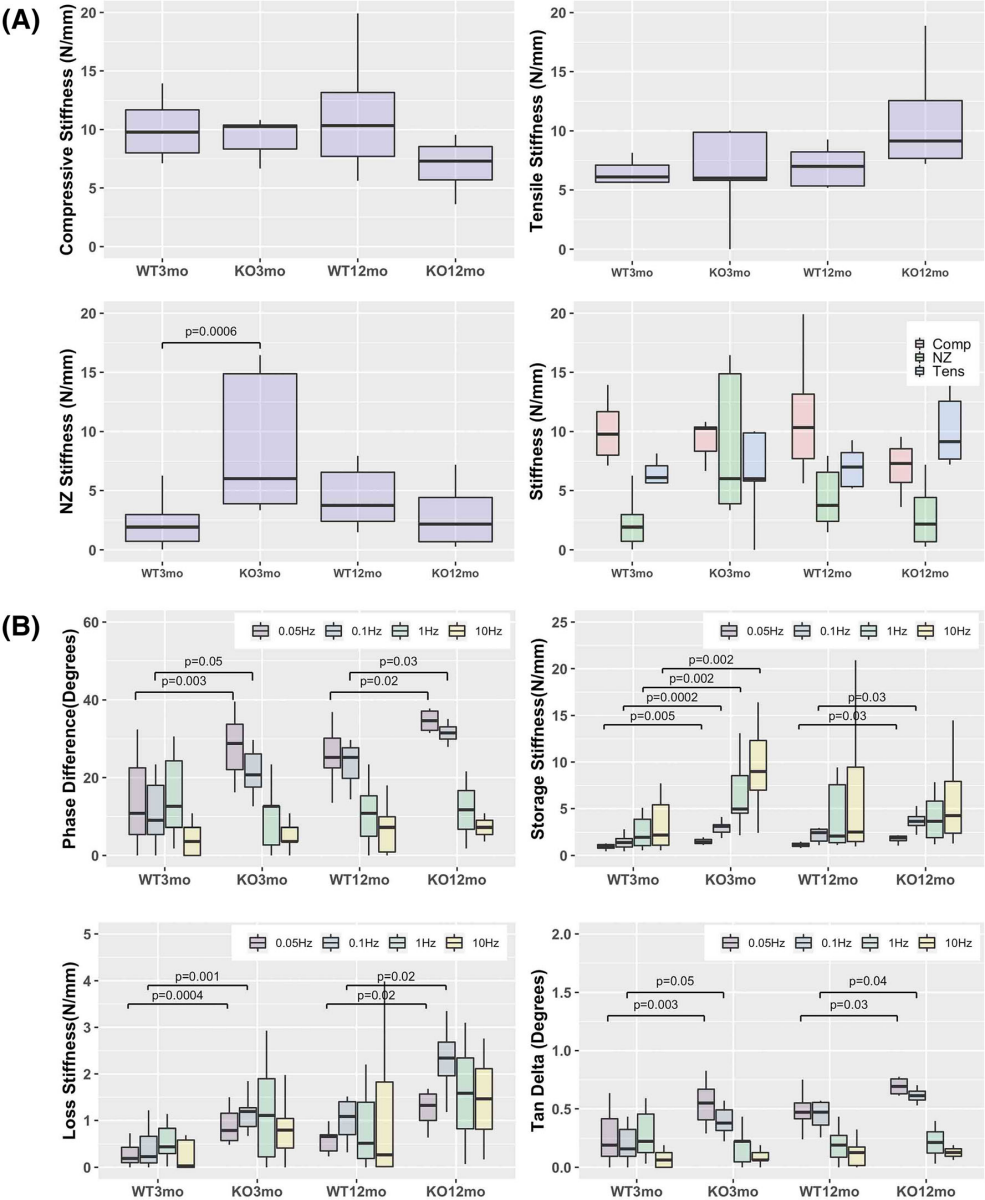
(A) Uninjured elastic biomechanical measures summary. No significant differences were seen between WT and IL‐1Ra−/− mice in most elastic measures. However, significant differences were seen between the IL‐1Ra−/− and WT mice at 3 months in NZ stiffness, but not in compressive or tensile stiffness (B) Uninjured Dynamic Mechanical Measures. As expected, phase angles were largest at the lower frequencies and smallest at the higher frequencies, in all groups. IL‐1Ra−/− mice had significantly higher phase angles at 0.05 and 0.1 Hz, with significant differences between 3‐month IL‐1Ra−/− vs WT, and also significant differences between 12‐month IL‐1Ra−/− vs WT. There were no differences in high‐frequency loading conditions (1 Hz and 10 Hz). Consequently, loss stiffness and tan delta were also significantly different between IL‐1Ra−/− and WT mice at lower frequency loading. Storage stiffness was different at all frequencies

There were no significant differences between IL‐1Ra−/− and WT mice in most elastic mechanical properties (Figure 2A). The sole exception was IL‐1Ra−/− mice which had a significantly stiffer NZ (6.6 ± 5.0 vs 2.3 ± 2.0 N/mm) (*p* < 0.02), and a much smaller NZ length (0.01 ± 0.04 vs 0.14 ± 0.03 mm) (*p* < 0.0001) compared to WT.

For dynamic mechanical measures, there were significant differences at the lower frequencies between IL‐1Ra−/− and the WT mice, at both 3 and 12 months but no differences at the higher frequencies (Figure 2B). For the 3‐month mice, the phase difference was much greater for the IL‐1Ra−/− group at 0.01 Hz (29.5° ± 10.5° vs 13.8° ± 10.8°) (*p* = 0.003) and 0.05 Hz (21.5° ± 5.9° vs 11.5° ± 8.6°) (*p* = 0.05). Similarly, for the 12‐month mice, the phase difference was significantly higher for the IL‐1Ra−/− group, at 0.01 Hz (34.7° ± 3.2° vs. 25.6° ± 7.6°) (*p* = 0.02) and 0.05 Hz (31.5° ± 3.0° vs. 23.7° ± 5.6°) (*p* = 0.03).

The significant phase shift difference at lower frequencies between IL‐1Ra−/− and WT mice led to significant differences in both 3‐ and 12‐month groups in storage stiffness, loss stiffness, and tan δ. Storage stiffness (E′, or the elastic component) was significantly higher for IL‐1Ra−/− IVDs at both 3 and 12 months. Loss stiffness (E″, or the viscous component) was similarly elevated in IL‐1Ra−/− IVDs. Tan delta (E″/E′) was significantly higher in 3‐month IL‐1Ra−/− IVDs.

#### 3.1.3 Molecular biology analysis—Uninjured IVDs

**FIGURE 3:**
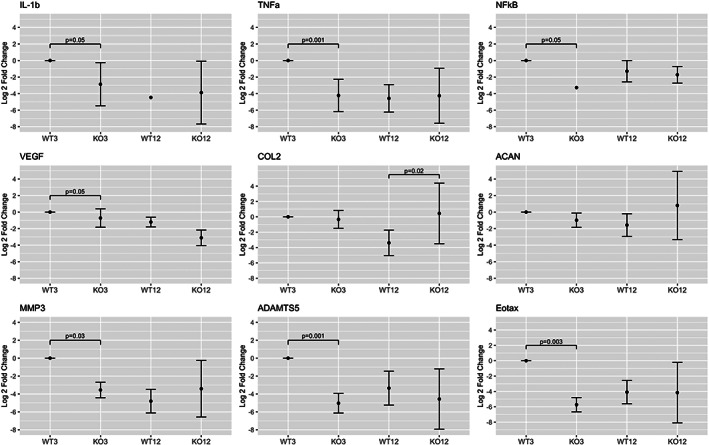
L‐1Ra−/− inflammatory, matrix anabolic and proteolytic gene expression from caudal IVDs. All inflammatory genes showed a statistically significant difference via ANOVA, especially between the 3‐month anchor and other groups. Inflammatory gene expression for IL‐1β, TNF‐α, and NF‐kB in 3‐ and 12‐month groups was much less in IL‐1Ra−/− mice compared to the 3‐month wild‐type anchor. For matrix anabolic genes, there were no differences at 3 months, while collagen II gene expression was significantly higher in IL‐1Ra ‐/‐ mice at 12 months than WT mice. For matrix proteolytic genes, MMP‐3 and ADAMTS‐5 gene expressions were lower in all groups when compared to the WT 3‐month group

Compared to WT controls at 3 months of age, IL‐1Ra−/− IVDs had strongly decreased expression of inflammatory and catabolic genes (Figure 3).

Compared to controls at 12 months, there was no appreciable difference in inflammatory gene expression between WT and IL‐1Ra−/− mice, nor for matrix proteolytic genes. The only significant difference was in COL2a1, where expression was significantly greater in the 12‐month IL‐1Ra−/− group compared to the 12‐month WT mice.

**FIGURE 4:**
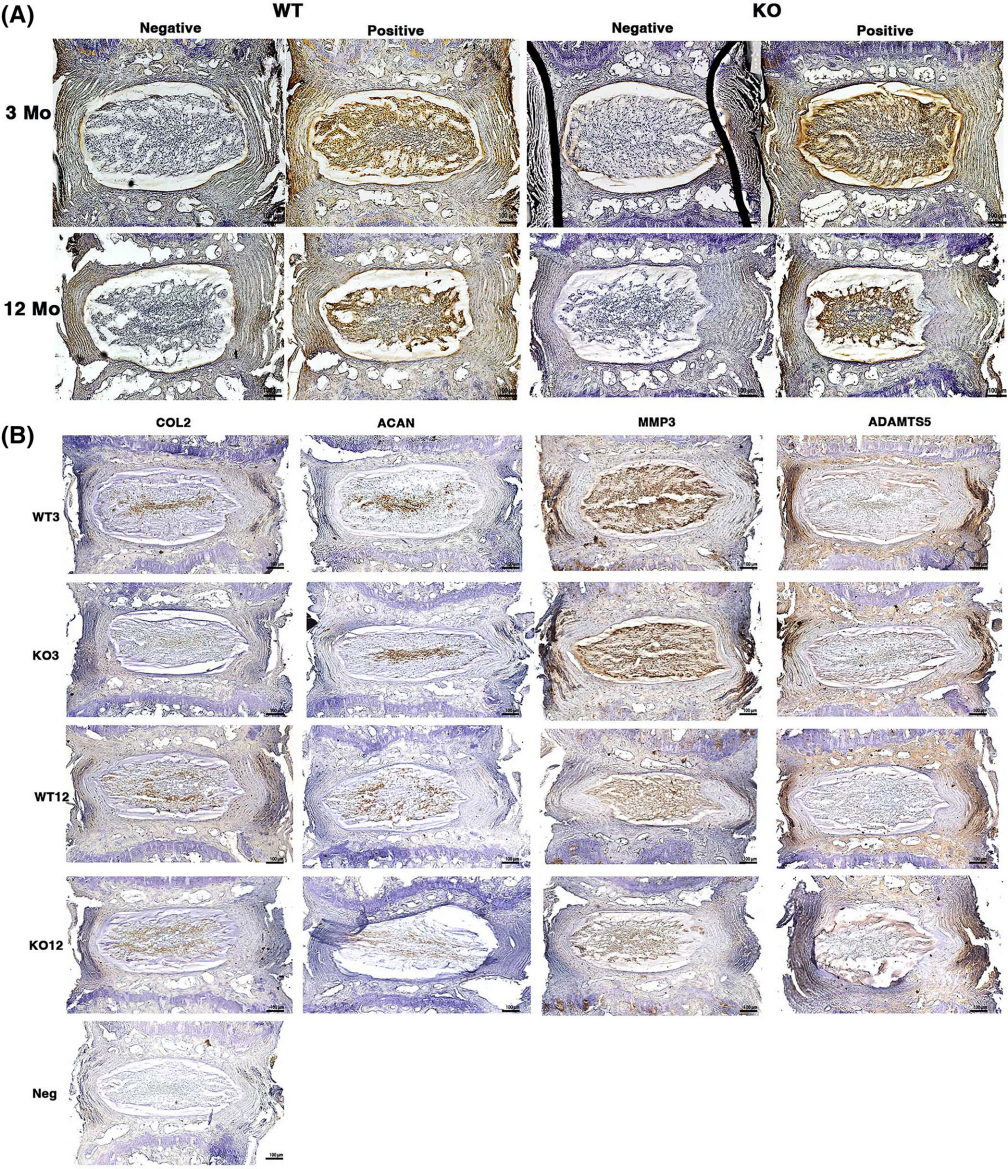
(A) Immunohistochemistry of IL‐1β and matrix proteins in uninjured caudal IVDs. IVDs underwent immunohistochemical staining with anti‐IL‐1β antibody and biotinylated secondary antibody, with HRP‐DAB (dark brown color) visualization. Intense IL‐1β staining was seen in the NP in most samples, with no difference between WT and IL‐1Ra−/−. (B) Immunohistochemistry of matrix anabolic proteins (Col2 and ACAN) and catabolic enzymes (MMP‐3 and ADAMTS‐5). Despite some differences in distribution between NP and AF for MMP‐3 (more in NP) and ADAMTS‐5 (more in AF), there were no differences between groups, even though there were gene expression differences

Immunolocalization of IL‐1β was intense in the NP in 3‐month IVDs of both WT (Figure 4A) and IL‐1Ra−/− mice and was similar in 12‐month IL‐1Ra−/− and older WT IVDs. Immunolocalization of matrix proteins (COL2, ACAN, MMP‐3, and ADAMTS‐5) was not different between 3‐month WT and IL‐1Ra−/− mice, nor between 12‐month WT and IL‐1Ra−/− mice (Figure 4B).

### 3.2 Injured IVDs

**FIGURE 5:**
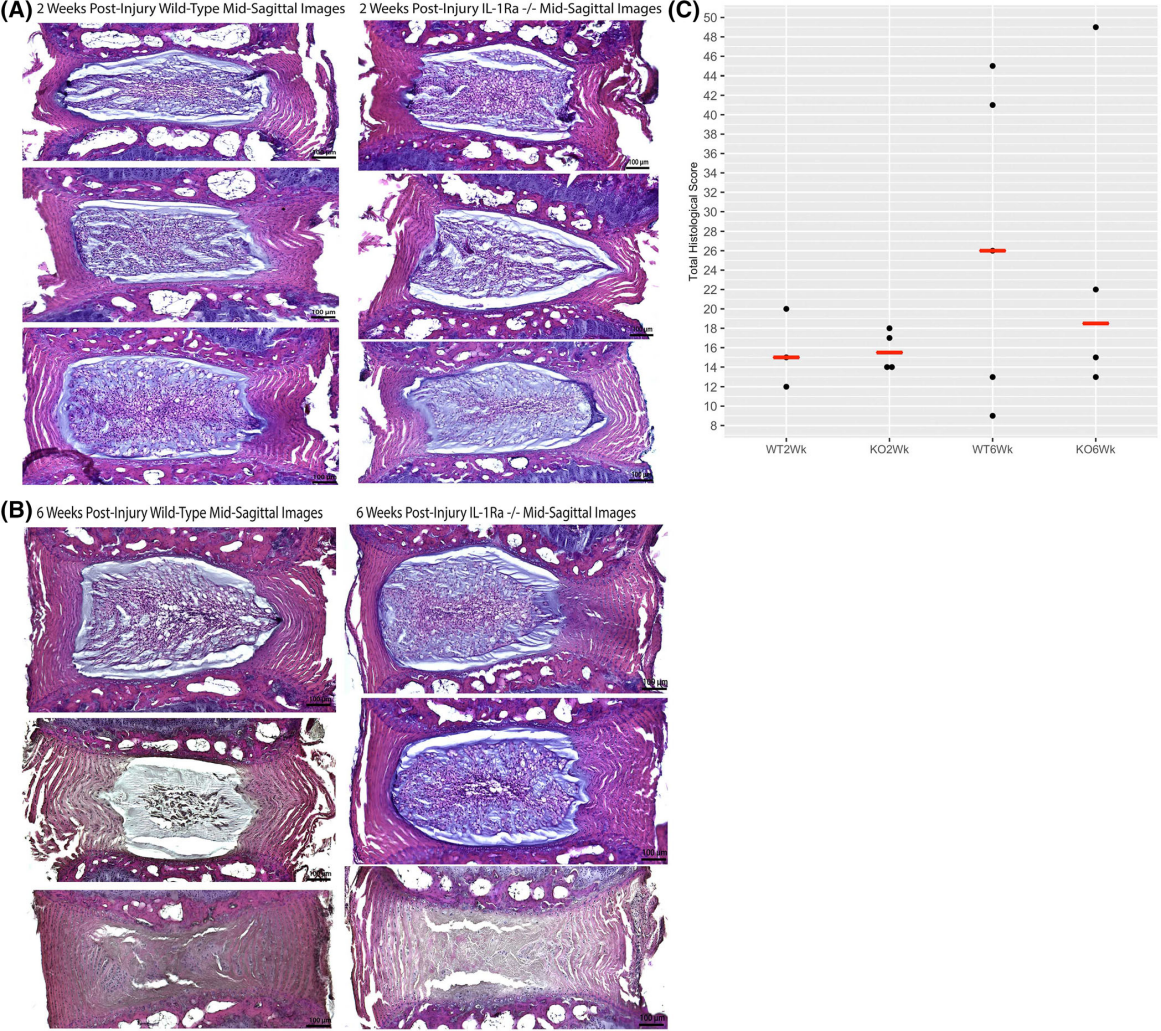
Injured caudal IVDs stained with H&E. Injured IVDs at 2 weeks (A) had significant annular morphological differences as compared to uninjured IVDs, but there were no significant differences between groups. At 6 weeks post injury (B), there was significant variation in magnitude of injury, but there were no differences between groups

IL‐1Ra −/− mice were smaller and shorter than WT mice. There was no difference in histological score between injured IVDs in WT and KO mice at 2 or 6 weeks post injury (Figure 5).

**TABLE 2:** Morphometric measurements of C57BL/6J IL‐1Ra−/− and wild‐type mice in annular puncture group

	2‐week KO (*n* = 11)	2‐week WT (*n* = 10)		6‐week KO (*n* = 10)	6‐week WT (*n* = 11)	
Torso length (mm)	82.9 (6.2)	89.3 (3.0)	*p* = 0.03	81.5 (2.7)	92.1 (4.5)	*p* = 0.001
Tail length (mm)	73.8 (3.5)	77.7 (1.7)	*p* = 0.02	78.2 (4.4)	76.0 (6.6)	
Body mass (g)	19.9 (5.6)	28.9 (5.3)	*p* = 0.004	20.0 (2.0)	31.9 (8.4)	*p* = 0.001

**FIGURE 6:**
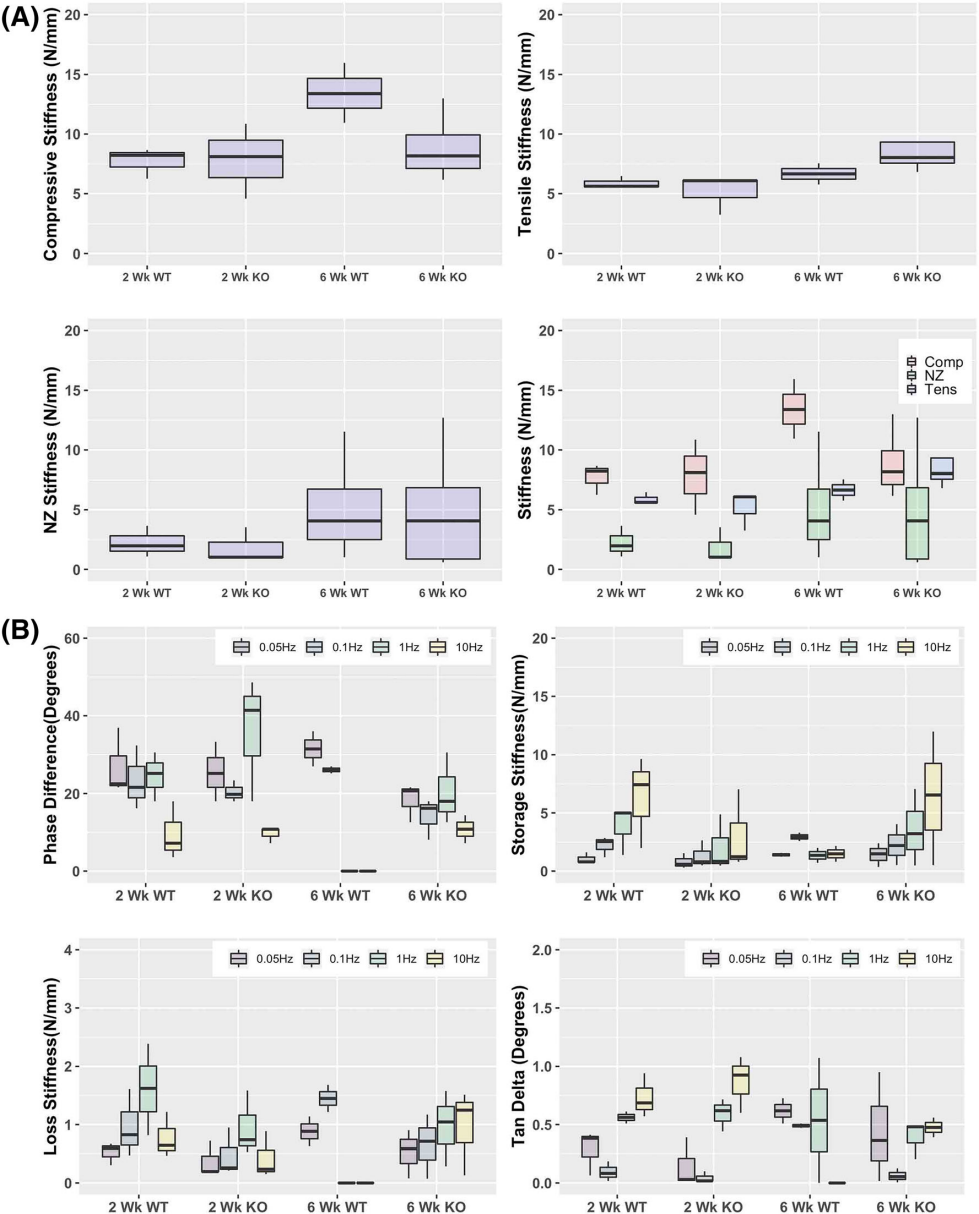
(A) Injured caudal IVD elastic biomechanical properties were not different between groups at 2 or 6 weeks post injury. (B) Dynamic mechanical properties were also not different between groups

There were no differences in compression, tension, or neutral zone stiffness between 2 weeks post injury of IL‐1Ra−/− and WT mice. There were no differences between genotypes at either the 2‐week or 6‐week mark in phase lag, which predicted no differences for any of the other dynamic measures (Figure 6).

**FIGURE 7:**
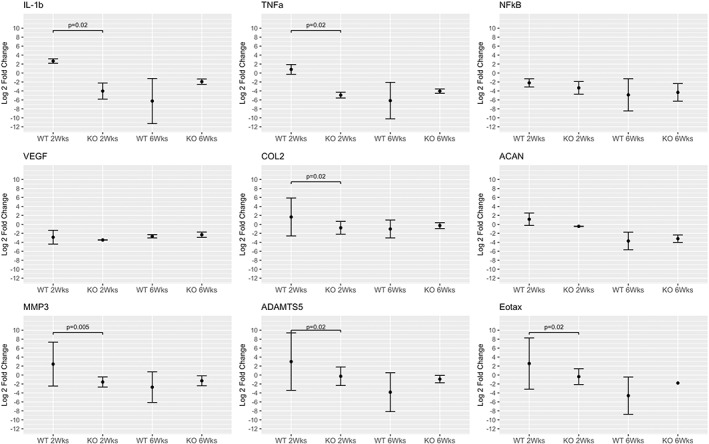
Inflammatory gene expression of injured wild‐type and IL‐1Ra−/− caudal IVDs at 2 and 6 weeks post injury. At 2 weeks post injury, there was significant upregulation of IL‐1β, TNF‐α, and eotaxin in WT mice, while there was significant downregulation of the same genes in IL‐1Ra−/− mice. At 6 weeks post injury, there was significant downregulation in both WT and IL‐1Ra−/− groups, as compared to the baseline condition of 3‐month uninjured WT mice; however, there was no difference between the WT and IL‐Ra−/− mice at 6 weeks post injury

At 2 weeks post injury, gene expression for inflammatory genes differed widely between WT and IL‐1Ra−/− post‐injured IVDs (Figure 7). There was significant *upregulation* of IL‐1β, TNF‐α, and eotaxin in WT mice, while there was significant *downregulation* of the same genes in IL‐1Ra−/− mice. In WT mice, IL‐1Ra expression did not increase. NF‐kB expression was decreased in both groups but was not significantly different.

ADAMTS5 and MMP3 expressions were significantly different between genotypes, mirroring IL‐1β gene expression differences, with a significant *upregulation* in WT mice, and a significant *downregulation* in IL‐1Ra−/− mice.

In 2‐week post‐injured WT mice, gene expression for collagen II and aggrecan expression was *upregulated* as compared to IL‐1Ra−/− mice, where gene expression for collagen II and aggrecan was significantly *decreased*.

At 6 weeks, there were no significant differences for any genes. All genes were substantially downregulated from baseline expression. IL‐1Ra gene expression in the WT group was significantly downregulated.

## 4 DISCUSSION

Although the initiating events of IVD degeneration remain obscure, pro‐inflammatory cytokines are active in the pathogenesis of IVD degeneration. It remains unclear whether pro‐inflammatory mediators are initiating factors or part of a biological response to injury or aging that promotes repair. While a variety of pro‐inflammatory cytokines have been identified in degenerating IVDs,^40^, ^41^ one of the most potent is IL‐1β.^8^ As the tissue half‐life of IL‐1β is rather short (<2 h^42^), much of our understanding of IL‐1β’s role in IVD homeostasis is derived from ex vivo and in vitro investigations. In most studies, IL‐1β is added at concentrations ranging from 5 to 20 ng/ml,^43^, ^44^, ^45^ while IL‐1β concentrations in vivo can be active at concentrations even below 1.75 ng/ml^46^ (i.e., 10^−10^ M). Phillips et al.^47^ performed a dose–response in vitro experiment stimulating cultured human disc herniation cells with IL‐1β in concentrations less than 1 ng, finding significant transcriptional upregulation of a wide variety of matrix proteins, cytokines, and chemokines even at this low dose. The present study reports on the structure and function of caudal IVDs in IL‐1Ra−/− C57BL/6J mice to test the effect of increased inflammatory activity induced by IL‐1Ra gene deletion in vivo.

As with any study, the present study has several limitations and assumptions as well as advantages. First, in a credible animal model of IVD degeneration with aging, IVDs should be normal in youth and develop progressive changes in structure and function with age. While the IVD seems to develop normally in this model, these mice had low body mass and a higher mortality rate. A tissue‐specific knockout mouse, driven by the Noto‐cre or other such promotor may have been a more suitable choice for a long‐term experiment. Nevertheless, IL‐1Ra−/− IVD on a C57BL/6J background exhibits relatively modest histological changes yet exhibits clear functional differences over a long latent period and represents a tractable murine IVD degeneration model for exploring anti‐inflammatory interventions. This model would need further mechanistic characterization before widespread use, including analysis of second messenger systems and cell death. As such, data presented here can be considered groundwork for future in vivo studies.

Second, the very small murine IVDs presented challenges for functional and biological testing. To reliably sample the whole caudal IVDs for qPCR analysis, a small wafer of epiphyseal bone was included in the IVD segment, which introduced a small contaminant of non‐IVD RNA in our samples. We performed an analysis comparing the gene expression of cells in the epiphyseal bone and cells in the IVD in young WT C57BL/6J mice (8 weeks of age) and have found higher levels of inflammatory gene expression in the epiphysis than in the IVD, while anabolic and catabolic matrix gene expression is higher in the IVD (Swamy 2019, unpublished). Therefore, while epiphyseal bone RNA may confound somewhat the *inflammatory* gene expression in this investigation, it has much less effect on IVD *matrix* gene expression.

Histopathological grading indicated that the degenerative changes seen in IL‐1Ra−/− IVDs were mainly driven by the scores of NP cellularity and NP matrix, with less obvious changes in AF or EP. A similar NP predominance of IL‐1β is seen in degenerative human IVDs, wherein AF (in particular outer AF) has little positive immunostaining for IL‐1β, even in degenerate samples.^8^ Some in vitro IVD studies have detailed a differential response to inflammatory activation between the AF and NP, with NP cells being much more responsive in terms of proteolytic gene expression.^48^ In the present study, this is confirmed by the spatial immunolocalization of IL‐1β primarily in the NP; hence, increased inflammation in IL‐1Ra−/− mouse seems localized primarily in the NP rather than the AF or EP.

The significant differences between IL‐1Ra−/− and WT mice in viscous measures, but not in elastic measures, are most consistent with the observed structural changes in the NP, rather than the AF. The mechanical changes of the murine IVD can be understood within the framework of a biphasic poroviscoelastic model, where responses to loading are governed by the interaction of a solid phase of tissue matrix and a fluid phase.^49^, ^50^ At high loading rates, mechanical behavior is primarily dependent on the initial pressurization of the fluid phase as well as matrix stiffness, resulting in a stiffer IVD response to load. At lower loading rates, fluid flow as well as solid‐phase creep has dominant effects. As rodent IVD is much more gelatinous and hydrated than adult human IVD, fluid flow is likely of heightened importance in its mechanical behavior.

In both the IL‐1Ra−/− and WT IVDs in this experiment, NPs were comprised mainly of notochordal cells, and thus, alteration in the mechanical properties of the notochordal cells themselves may account for the observed differences in dynamic mechanical analysis. Remarkably, when the mechanical properties of individual IVD cells are measured by micropipette aspiration techniques, notochordal NP cells are much stiffer than AF cells, and 1.5 to 2 times stiffer than estimates for human NP matrix.^51^ Furthermore, inflammation can directly change cellular mechanical properties. When cultured bovine NP cells are exposed to an inflammatory challenge, cell permeability and cell radius significantly increase, with a concomitant increase in IL‐1β gene expression.^52^ While speculative, there is a plausible link between inflammation, notochordal cell permeability, and bulk mechanical properties in the rodent IVD, which is a testable hypothesis.

While cellular biophysical changes might account for the observed differences in IVD mechanical properties, it seems more plausible that progressive matrix loss and IVD catabolism secondary to unchecked inflammation are more important. The premise of the present study was that unchecked IL‐1β activity in IVD in the absence of its natural competitive antagonist IL‐1Ra would increase inflammatory gene expression (TNF‐α and NF‐kB)^53^ and matrix catabolic gene expression.^54^ However, we observed the opposite in 3‐month IL‐1Ra−/− mice: inflammatory gene expression fell in the IL‐1Ra−/− groups, and matrix proteolytic gene expression fell in concert, while matrix anabolic gene expression seemed unaltered. A similar gene expression pattern was observed after an annular puncture. As protein and activity levels of proteolytic enzymes and degradation products of matrix constituents were not directly measured, the relationship between the gene expression and matrix function is indeterminate. Nonetheless, a decrease in inflammatory gene expression in the IL‐1Ra−/− mouse is expected, given that circulating levels of IL‐1β in serum of IL‐1Ra−/− C56BL mice are reportedly near half those of the WT.^55^ Hence, our findings are consistent with those of others in this mouse.

Synchronized IL‐1Ra and IL‐1β gene expression levels suggest co‐regulation of the agonist and its competitive antagonist can occur, and various mechanisms have been proposed. For example, IL‐1Ra and IL‐1β have similar transcriptional regulation, which is perhaps unsurprising given that both molecules are produced in response to the same inflammatory signals (TNF‐α, LPS, and IL‐1),^56^ are activated by Toll‐like receptors,^57^ and share NF‐kB elements in promoter regions.^58^ While transcription mechanisms exist to account for IL‐1Ra and IL‐1β co‐expression,^57^ these have not been well‐studied or described in the IVD or other cartilaginous tissues. Further study of gene networks in vivo will likely further clarify and confirm the role of IL‐1β in IVD function. Indeed, low levels of IL‐1β‐related inflammation may be necessary for matrix homeostasis.

Interstrain differences in the IL‐1Ra−/− mouse are known and likely account for the observed differences in IVD degeneration. Specifically, the IL‐1Ra−/− mouse on a C57BL/6J background has a low prevalence of baseline inflammatory arthritis, yet when backcrossed to a BALB/c mouse, spontaneous arthritis is seen in approximately 80% of mice by 8 weeks of age.^13^ More recently, inflammatory arthritis in these mice reportedly depends on increased responsiveness of the 𝛾δ T‐cell to IL‐1β stimulation, which is seemingly resistant to the antagonistic moderating effects of IL‐1Ra.^14^

The effect of the IL‐1Ra gene deletion on IVD has been reported on a BALB/c background.^15^ IVDs in this study exhibited profound changes, with severe degeneration by 2 months, followed by complete loss of IVD architecture and ossification by 6 months.^15^ In the present study, the natural history of IL‐1Ra−/− on a C57BL/6J background includes much less severe changes to the IVD, which occur over a longer interval. BALB/c IL‐1Ra−/− mice had very high expression of IL‐1β protein in their IVDs,^15^ while we found no significant increases on a C57BL/6J background; differential IL‐1β expression in the IVD could explain the aggressive degeneration seen on the BALB/c background. Further clarification of interstrain differences in IVD of the IL‐1Ra−/− mouse would benefit from further characterization of the innate immune system, which would be informative for modeling IVD degeneration and inflammatory diseases of the IVD.

This present investigation is an initial characterization of the in vivo effect of alteration of the IL‐1β system in IVD in C57BL/6 mice. We observed that while IVD of IL‐1Ra−/− mice exhibits subtle histological changes compared to WT mice, they show marked differences in function (biomechanics); that is, despite the structural similarity, they are functionally different. We speculate that the co‐regulation of inflammatory, matrix production, and matrix proteolytic gene expression in IVD represents a homeostatic mechanism for maintaining disc health. Uncoupling of homeostasis in IVD may tip the balance toward catabolism and degeneration. In the context of these studies, the unchecked activity of the pro‐inflammatory cytokine IL‐1β can significantly affect IVD structure and function over the lifetime.

## AUTHOR CONTRIBUTIONS

*Collection of data, design, data analysis, writing, and editing the paper*: Ganesh Swamy and John Matyas. *Design, proofreading, and editing*: Paul Salo, Frank Jirik, and John Matyas. *Design, data analysis, proofreading, and editing*: Neil Duncan.

## Supporting information


Tables S1 and S2
