# Supplementary material for: IL‐1Ra deficiency accelerates intervertebral disc degeneration in C57BL6J mice
Source: JOR Spine. 2022 Apr 23;5(2):e1201. doi: 10.1002/jsp2.1201 (PMC9238285; doi:10.1002/jsp2.1201)
Supplement: Supplementary file 1 — Tables S1 and S2 [file JSP2-5-e1201-s001.docx]

Supplementary Table 1

| **Gene** |  | **NCBI geneid** | **Forward Primer (5' to 3')** | **Reverse Primer (5' to 3')** | **Amplicon Size** | | **PrimerbankID** |
| --- | --- | --- | --- | --- | --- | --- | --- |
| **IL-1b** | Il1b | 16176 | GCAACTGTTCCTGAACTCAACT | ATCTTTTGGGGTCCGTCAACT | | 89 | 6680415a1 |
| **TNFa** | Tnf | 21926 | CCCTCACACTCAGATCATCTTCT | GCTACGACGTGGGCTACAG | | 61 | 7305585a1 |
| **IL-1Ra** | Il1rn | 16181 | GCTCATTGCTGGGTACTTACAA | CCAGACTTGGCACAAGACAGG | | 132 | 13624317a1 |
| **NF-kB (p105)** | Nfkb1 | 18033 | ATGGCAGACGATGATCCCTAC | TGTTGACAGTGGTATTTCTGGTG | | 66 | 200217a1 |
| **Col2** | Col2a1 | 12824 | GGGAATGTCCTCTGCGATGAC | GAAGGGGATCTCGGGGTTG | |  |  |
| **Aggrecan** | Acan | 11595 | CCTGCTACTTCATCGACCCC | AGATGCTGTTGACTCGAACCT | | 150 | 6671523a1 |
| **MMP3** | Mmp3 | 17392 | TTAAAGACAGGCACTTTTGGCG | CCCTCGTATAGCCCAGAACT | | 170 | 6754713c2 |
| **ADAMTS5** | Adamts5 | 23794 | GGAGCGAGGCCATTTACAAC | CGTAGACAAGGTAGCCCACTTT | | 110 | 6752976a1 |
| **VEGF** | Vegfa | 22339 | GCACATAGAGAGAATGAGCTTCC | CTCCGCTCTGAACAAGGCT | | 105 | 6678563a1 |
| **Eotaxin** | Ccl11 | 20292 | GAATCACCAACAACAGATGCAC | ATCCTGGACCCACTTCTTCTT | | 98 | 6755418a1 |
| **B2M** | B2m | 12010 | TTCTGGTGCTTGTCTCACTGA | CAGTATGTTCGGCTTCCCATTC | | 104 | 31981890a1 |
|  |  |  |  |  | |  |  |

Supplementary Table 2

Histological Scoring for Murine IVD Degeneration (Modified Masuda / Rutges Scale)

| A. Endplate | | | |  | |  | |  | |  | |  | |  | |
| --- | --- | --- | --- | --- | --- | --- | --- | --- | --- | --- | --- | --- | --- | --- | --- |
|  | 0 | Homogeneous structure; regular thickness | | | | | | |  | |  | |  | |  |
|  | 1 | Slight irregularity with microfractures and locally decreased thickness | | | | | | | | | | |  | |  |
|  | 2 | Severe irregularity; multiple microfractures and generalized decreased thickness | | | | | | | | | | | | |  |
|  |  |  |  | |  | |  | |  | |  | |  | |  |
| B. Morphology AF | | |  | |  | |  | |  | |  | |  | |  |
|  | 0 | Well-organized, half ring-shaped structure, collagen lamellae | | | | | | | | |  | |  | |  |
|  | 1 | Partially ruptured AF; loss of half ring-shape | | | | | | | | |  | |  | |  |
|  | 2 | Completely ruptured AF; no intact half ring-shaped collagen lamellae | | | | | | | | | | |  | |  |
|  |  |  |  | |  | |  | |  | |  | |  | |  |
| C. Boundary AF / NP | | |  | |  | |  | |  | |  | |  | |  |
|  | 0 | Clear boundary | | |  | |  | |  | |  | |  | |  |
|  | 1 | Boundary less clear | | |  | |  | |  | |  | |  | |  |
|  | 2 | No boundary | | |  | |  | |  | |  | |  | |  |
|  |  |  |  | |  | |  | |  | |  | |  | |  |
| D. Cellularity NP | | |  | |  | |  | |  | |  | |  | |  |
|  | 0 | Normal cellularity - large vacuoles in the gelatinous structure of the matrix | | | | | | | | | | |  | |  |
|  | 1 | Mixed cellularity - normal pattern; some clusters; partial loss of notochordal cells | | | | | | | | | | | | |  |
|  | 2 | Moderate/severe decrease in the number of cells and no notochordal cells | | | | | | | | | | |  | |  |
|  |  |  |  | |  | |  | |  | |  | |  | |  |
| E. Matrix NP | | |  | |  | |  | |  | |  | |  | |  |
|  | 0 | Normal gelatinous appearance; well-organized structure | | | | | | | | |  | |  | |  |
|  | 1 | Partially disorganized structure of matrix | | | | | | |  | |  | |  | |  |
|  | 2 | Complete disorganization and loss/condensation of matrix | | | | | | | | |  | |  | |  |
